# Etiologies et pronostic des occlusions intestinales aigues mécaniques à l’Hôpital National de Zinder: étude transversale sur 171 patients

**DOI:** 10.11604/pamj.2016.24.248.8372

**Published:** 2016-07-15

**Authors:** Harissou Adamou, Ibrahim Amadou Magagi, Oumarou Habou, Amadou Magagi, Halidou Maazou, Mansour Adamou, Yacouba Harouna

**Affiliations:** 1 Service de Chirurgie Générale et Digestive B, Hôpital National de Zinder, Niger; 2 Service de Chirurgie Pédiatrique, Assistant à la Faculté des Sciences de la Santé de Zinder, Niger; 3 Service d’Anesthésie-Réanimation, Hôpital National de Zinder, Niger; 4 Service d’Urologie, Hôpital National de Zinder, Niger; 5 Service de Chirurgie Viscérale C, Hôpital National de Niamey, Niger

**Keywords:** Occlusions intestinales, étiologies, pronostic, urgences, chirurgie, hernies étranglées, Intestinal obstruction, etiology, prognosis, emergency, surgery, strangulated hernias

## Abstract

L’occlusion intestinale aigue (OIA) mécanique, représente l’une des pathologies les plus fréquentes en chirurgie digestive d’urgence. L’objectif de cette étude était de décrire les aspects étiologiques et pronostiques des occlusions intestinales aigues mécaniques à l’Hôpital National de Zinder (HNZ), Niger. Il s’agissait d’une étude transversale sur 24 mois (Janvier 2013 à Décembre 2014) ayant inclus tous les patients opérés pour OIA. Les occlusions intestinales mécaniques représentaient 24,50% (n=171) des urgences chirurgicales digestives (n=622). L’âge médian était à 25 ans (extrêmes: 1 jour et 95 ans). Le sexe ratio était à 3,5 en faveur des hommes. Les enfants représentaient 38,60% (n=66). Le siège de l’obstacle était sur le grêle dans 60,82% (n=104), colique dans 21,63%(n=37) et mixte dans 17,54% (n=30). Le mécanisme par strangulation constituait 88,89% (n=152) dont les hernies étranglées avec 49,70% (n=85) et les invaginations intestinales aigues avec 19,88% (n=34) des patients. Les malformations ano-rectales et les tumeurs constituaient les principales étiologies par obstruction avec respectivement 7,02% (n=12) et 3,51% (n=6). Une résection intestinale était faite dans 52 cas (30,41%). Les complications septiques prédominaient (n=39/53), dont la suppuration pariétale (n=23). Le séjour moyen était de 7,82 jours. La mortalité globale était 11,70% (n=20). Celle-ci était corrélée statistiquement à la nécrose intestinale (p=0,01) et au retard d’admission (p=0,04). Les étiologies des OIA sont multiples et dominées par les hernies étranglées. La morbi-mortalité élevée qu’elles entrainent pourrait être évitée par la prise en charge précoce avant l’installation d’une nécrose intestinale.

## Abstract (en)

Acute mechanical intestinal obstruction (AIO) is one of the most frequent pathologies in emergency digestive surgery. The objective of this study was to describe the etiologic and prognostic features of acute mechanical intestinal obstruction at the National Hospital of Zinder (HNZ), Niger. We conducted a cross-sectional study including all the patients operated for AIO over a period of 24 months (January 2013 - December 2014) Mechanical intestinal obstructions represent 24.50% (n=171) of digestive surgical emergencies (n=622). The median age was 25 years (range: 1 day-95 years). The sex ratio was 3.5 in favour of men. Children accounted for 38.60% (n=66). The seat of the obstacle was at the level of the small bowel in 60.82% (n=104), colonic in 21.63% (n=37) and mixed in 17.54% (n=30). The mechanism of strangulation accounted for 88,89% (n=152), among these strangulated hernias were detected in 49,70% (n=85) of patients and acute intestinal invaginations in 19,88% (n=34) of patients. Anorectal malformations and tumors were the main cause of obstruction in 7.02% (n=12) and 3.51% (n=6) of cases respectively. Intestinal resection was performed in 52 cases (30.41%). Septic complications prevailed (n=39/53); among these parietal suppuration (n=23). The average length of stay in hospital was 7.82 days. The overall mortality rate was 11.70% (n=20). This was statistically correlated to intestinal necrosis (p=0.01) and to delayed hospital admission (p=0.04). There are many causes of AIO which are dominated by strangulated hernia. The high morbidity and mortality rate from AIO might be prevented by early treatment performed before intestinal necrosis occurs.

## Introduction

Les occlusions intestinales aigues mécaniques, correspondent un arrêt complet et persistant du contenu intestinal par strangulation ou obstruction [1–3]. Elles représentent l’une des principales urgences en chirurgie digestive [1, 2, 4–7]. Elles nécessitent un diagnostic précoce et une prise en charge adéquate. Leurs étiologies restent multiples et varient en fonction des pays ou de l’âge [2, 3, 5, 8–13]. Le pronostic dépend de la survenue d’une nécrose intestinale et de l’efficacité de la prise en charge [2, 3, 5, 7, 8, 11, 14–17]. L’objectif de cette étude était de décrire les aspects étiologiques et pronostiques des occlusions intestinales aigues mécaniques à l’Hôpital National de Zinder (HNZ) au Niger.

## Méthodes

Il s’agissait d’une étude transversale sur une période de 24 mois (Janvier 2013 à Décembre 2014) ayant concerné tous les patients opérés pour occlusion intestinale mécanique à l’Hôpital National de Zinder-Niger. Etaient exclus les patients ayant une occlusion intestinale fonctionnelle et les autres urgences digestives.

Les variables suivantes étaient étudiées: l’âge, le sexe, la provenance, le mode d’admission, le délai entre l’apparition des premiers signes et l’arrivée à l’hôpital, le délai passé à l’hôpital avant l’intervention chirurgicale, les gestes chirurgicaux réalisés, les étiologies, les complications postopératoires, la durée du séjour hospitalier et le mode de sortie des patients. Les données recueillies des dossiers des patients et des différents registres services ont été enregistrées et analysées avec les logiciels Excel 2003 et Epiinfo7. Les tests t de Student et de Kruskal-Wallis, ont été utilisés pour comparer les proportions ou les moyennes. Le test de Khi2 pour faire des associations qualitatives. Le seuil de signification statistique retenu pour les tests était de 5%.

## Résultats

Durant la période d’étude, 622 cas d’urgences chirurgicales digestives ont été pris en charge. Les occlusions intestinales mécaniques représentaient 24,50% (n=171). Les patients étaient admis sans référence dans 61,99% (n=106) et 67,25% (n=115) provenaient du milieu rural. L’âge médian était de 25 ans (extrêmes: 1 jour et 95 ans). Le sexe ratio était de 3,5 en faveur des hommes. Les enfants représentaient à 38,60% (n=66) et 57,89% des patients étaient âgés de moins de 30 ans (Tableau 1).

**Tableau 1:** Répartition des patients par tranche d’âge, par sexe et nombre de décès

Groupe d’âge (ans)	SEXE		Nombre (%)	Nombre de décès
	Féminin	masculin		
[0-15[	19	47	66 (38,60)	12
[15-30]	6	27	33 (19,30)	2
]30-45]	2	30	32 (18,71)	4
]45-60]	8	16	24 (14,03)	1
Plus de 60	3	13	16 (9,35)	1
**Total**(%)	**38**(22,22%)	**133**(77,78%)	**171**(100)	**20**

Le délai moyen d’admission à partir du début de la symptomatologie était de 47,61 heures (extrêmes: 2 à 168 heures). Le siège de l’obstacle était sur le grêle dans 60,82% (n=104), colique dans 21,63%(n=37) et mixte dans 17,54% (n=30). La strangulation constituait le principal mécanisme de survenue des occlusions soit 88,89% (n=152). Dans ce groupe les hernies étranglées étaient retrouvées dans 49,70% des cas (n=85) et les invaginations intestinales aigues (IIA) dans 19,88% (n=34). Les malformations ano-rectales et les tumeurs constituaient les principales étiologies par obstruction avec respectivement 7,02% (n=12) et 3,51% (n=6) (Tableau 2).

**Tableau 2:** Répartition étiologique des patients

Mécanismes n (%)	Causes		n (%)	Mode de sortie	
				Amélioré	Décédé
Strangulation n=152 (88,89%)	***Hernies étranglées (n=85)*** *(Age moyen : 33,70 ans)*	Inguinales	53 (31)	50	3
		Ombilicales	24 (14,04)	24	0
		épigastrique	1 (0,58)	1	0
		Crurales	5 (2,92)	5	0
		Internes+	2 (1,17)	1	1
	***Invagination intestinale aigue*** *(Age médian=4ans)*		34 (19,88)	28	6
	***Occlusion sur brides*** *(Age moyen : 37,14 ans)*		22 (12,87)	20	2
	***Volvulus du sigmoïde*** *(Age moyen : 47,54 ans)*		11 (6,43)	9	2
Obstruction n=19 (11,11%)	***Malformations coliques et ano-rectales***	Imperforation anale	12 (7,02)	8	4
		Atrésie colique	1 (0,58)	1	0
	***Tumeurs colorectales***		6 (3,51)	4	2
**Total (%)**			**171 (100%)**	**151 (88,30%)**	**20(11,70%)**

+ une hernie interne supravésicale et une hernie diaphragmatique étranglées

L’âge moyen pour les hernies étranglées était de 33,70 ans. Dans ce groupe, les hernies inguinales représentaient 62,35% (53/85) des cas. La majorité des invaginations intestinales aigues étaient retrouvées chez l’enfant (30/34) et le nourrisson (21/34); l’âge médian était à 4 ans (extrêmes 3 mois et 50 ans). L’IIA était noté chez 4 adultes dont 2 cas de tumeurs du grêle. Pour les cas du volvulus du sigmoïde, la moyenne d’âge était de 47,54 ans (Tableau 2). La nécrose intestinale était retrouvée chez 30,41% (n=52) des patients. Ce qui a conduit à une résection intestinale segmentaire avec anastomose en un temps pour 28,65% (n=49) des patients. Le délai médian d’intervention était de 8 heures (extrêmes: 2 à 28 heures). Le (Tableau 3) résume les différents gestes chirurgicaux réalisés.

**Tableau 3:** Principaux gestes chirurgicaux

Intervention chirurgicale (n)	Localisation anatomique		Nombre (%)
Herniorraphie simple			62 (36,26)
Résection intestinale (n=52)	Résection grêle	Anastomose en 1 temps	33 (19,30)
		Iléostomie	3 (1,75)
	Colectomies	Hémicolectomie droite	12 (7,02)
		Selon Hartmann	4 (2,34)
Stomies digestives	Colostomie temporaire	Transverse	12 (7,02)
		Sigmoïdostomie	7 (4,09)
	Colostomie définitive	Colostomie iliaque	4 (2,34)
Résection brides et adhésiolyse			14 (8,19)
Désinvagination			20 (11,70)
**Total**			**171 (100)**

La morbidité était de 31% (n=53). Les complications septiques prédominaient (n=39) avec en tête, la suppuration pariétale (n=23), suivi du choc septique (n=12), des péritonites postopératoires (n=2) et des éviscérations (n=2). Nous avions noté 12 cas d’anémie (<10 g/dl), 1 cas de détresse respiratoire et 1 cas d’embolie pulmonaire.

La durée moyenne de séjour était de 7,82±4,01 jours (extrêmes: 1 à 45 jours). La mortalité globale était 11,70% (n=20). Celle-ci était corrélée statistiquement à la nécrose intestinale (p=0,01). Cette nécrose était plus fortement associée à la strangulation (p=0,01) qu’au retard d’admission (p=0,04). Le taux de décès double (21,15%) pour les patients (11/52) ayant présenté une nécrose. La mortalité pour les imperforations anales et les tumeurs colorectales était de 33,33% (Tableau 2). La mortalité dans la population de 0 à 15 ans était de 18,18% (12/66) (Tableau 1).

## Discussion

Durant la période de cette étude, l’occlusion intestinale aigue (OIA) occupait la 2ème place des urgences chirurgicales digestives à l’Hôpital National Zinder avec 24,5%. Cette fréquence importante de l’OIA en chirurgie digestive d’urgence a déjà été notée dans plusieurs études [1, 2, 4, 5, 10, 15]. La majorité de nos patients étaient jeunes (57,90% avaient un âge < 30ans et 76,61% avaient moins de 45ans) et de sexe masculin (sex ration=3,5). Dans la plupart des études (14-16) en milieu subsaharien, l’OIA étaient plus fréquente chez l’enfant ou l’adulte jeune. En Europe Markogianakis et al [2] ayant inclus des patients âgés d’au moins 14 ans, avaient trouvé un âge moyen de 63,8 ans.

Dans notre échantillon, le siège de l’occlusion était dans 60,82% sur le grêle et 21,63% colique. Comme beaucoup d’auteurs l’avaient rapporté déjà, l’obstacle siège souvent sur le grêle [1, 7]. Contrairement à plusieurs études [7, 12, 15, 16] qui classent les brides et adhérences postopératoires en tête des mécanismes d’OIA par strangulation, au cours de notre étude, ce sont les étranglements herniaires et les invaginations intestinales qui constituaient les deux premières causes; les brides ne représentaient que 12,87%.

En occident, les brides et les adhérences postopératoires constituent la première cause [8]. Markogianakis et al [2], Jackson et al [3], notaient respectivement 73,8% et 60% d’ occlusions par adhérence. Pour Cappell et al [5] 75% d’occlusion du grêle sont dues à des adhérences postopératoires. Dans plusieurs pays développés et dans certains pays en développement, la prise en charge chirurgicale précoce des hernies simples non compliquées contribue à réduire significativement les occlusions par étranglement herniaire [5, 7, 16]. Dans notre contexte nigérien, les hernies étranglées constituent encore la première cause d’OIA mécaniques. Il y a plus de 10 ans, Harouna et al à Niamey [10], notaient que 60% des étiologies d’occlusions intestinales aigues étaient des hernies étranglées. En effet, les patients consultaient souvent lorsque la hernie est étranglée et arrivent aussi en retard. Ce qui conduit à la nécrose intestinale; facteur de mauvais pronostic [14].

Les invaginations intestinales aigues (IIA) représentaient la 2^ème^ cause des OIA par strangulation dans notre série avec 19,88% (n=34). L’IIA est l’étiologie des OIA la plus fréquemment retrouvée chez l’enfant et le nourrisson. Elle est idiopathique dans 90% des cas [13, 17–20]. Elle est rare chez l’adulte et souvent liée à une cause organique [21–23]. Les malformations ano-rectales constituaient 7,02% de nos patients. Pour Homawoo et al [17], les imperforations anales représentaient 8,10% des causes d’occlusions en milieu pédiatrique.

Chez nos patients, les causes d’occlusions coliques étaient dominées par les invaginations, les volvulus du sigmoïde et les malformations ano-rectales. Les tumeurs colorectales ayant entrainé une occlusion colique étaient moins fréquentes (3,51%) dans notre série, contrairement aux données occidentales: Jackson et al [3] 20%, Markogianakis et al [2]: 47,4%, Cappell et al [5]: 60%. Le volvulus du sigmoïde représentait dans notre série 6,43% des causes d’OIA. Le volvulus du sigmoïde est plus fréquent en Afrique qu’en occident [10, 11, 24]. Pour Ohene-Yeboah et al [11], Harouna et al [10], avaient enregistré respectivement dans leur série, une fréquence du volvulus du colon sigmoïde à 5,83%, 13,3% des OIA mécaniques. Dans l’étude de Traoré et al [24], le volvulus du sigmoïde représentait 2,4% des urgences chirurgicales.

Dans notre étude, la nécrose intestinale a été retrouvée chez 30,41% et a conduit à une résection intestinale avec anastomose termino-terminale en un temps ou suivi d’une stomie. Les complications postopératoires étaient enregistrées chez 31% des patients et les infections étaient à 22,80%. Notre taux de décès global était de 11,70%. Harouna et al [14] avaient noté une résection intestinale justifiée par l’existence d’une nécrose dans 37% des cas et une lourde mortalité postopératoire à 34,5%. Cette mortalité très élevée était liée à l’âge, la strangulation et la nécrose intestinale, les retards d’admission et du traitement. Ohene-yeboah et al [11] notaient un taux de décès global à 12%. Malik et al [7] et Ooko et al [15] avaient noté une mortalité globale plus basse de 3,49% et 4,5%. La mortalité était statistiquement corrélée à la nécrose intestinale (p= 0,01). Ce qui confirme les travaux de plusieurs études sur les occlusions intestinales aigues [5, 10, 15, 24].

## Conclusion

Les étiologies des OIA sont multiples et dominées par les hernies étranglées. La morbi-mortalité élevée qu’elles entrainent pourrait être évitée par la prise en charge précoce avant l’installation d’une nécrose intestinale. Ceci justifie dans notre contexte, une amélioration de la couverture sanitaire en rendant prioritaire les soins chirurgicaux essentiels.

### Etat des connaissances actuelles sur le sujet

- Les occlusions intestinales aigues mécaniques sont des urgences chirurgicales digestives fréquentes avec une diversité d’étiologies;
- Les causes restent dominées par les brides et les adhérences postopératoires.

### Contribution de notre étude à la connaissance

- Dans le sud-est du Niger, les hernies étranglées constituent la première cause des occlusions intestinales aigues mécaniques;
- Dans notre contexte, la nécrose intestinale fréquente est un facteur de mauvais pronostic;
- L’accès au traitement chirurgical des hernies simples, avant l’étranglement demeure encore un challenge dans le système de santé au Niger.
